# Increase in the wave power caused by decreasing sea ice over the Sea of Okhotsk in winter

**DOI:** 10.1038/s41598-023-29692-9

**Published:** 2023-02-13

**Authors:** Shinsuke Iwasaki

**Affiliations:** Civil Engineering Research Institute for Cold Region, Public Works Research Institute, 1–3–1–34, Toyohira, Sapporo, 062–8602 Japan

**Keywords:** Physical oceanography, Climate-change impacts

## Abstract

Increasing ocean surface waves owing to decreasing sea ice in the Sea of Okhotsk (SO) is a major concern. However, long-term trends of ocean surface waves in the SO have not yet been investigated. Therefore, the long-term trends of wave power (${P}_{\mathrm{w}}$) in the SO were investigated using the 40-year (from the 1980s) simulations and based on the wave model (WAVEWATCH III) enforced by three reanalyses and one satellite product. Three wave model simulations were conducted using the original (hourly or daily) reanalysis or satellite sea ice data. In addition, to quantify the responses of the long-term ${P}_{\mathrm{w}}$ trends to surface winds and sea ice, three wave model simulations were performed with climatological sea ice data, for a total of six model simulations. The model results of the original sea ice data corresponded well with the buoy observations. Moreover, ${P}_{\mathrm{w}}$ increased (~ 12–15% per decade) remarkably during winter (December–February). The increased ${P}_{\mathrm{w}}$ could be attributed to the strengthened surface winds and reduced sea ice (i.e., reduction of direct wave decay by sea ice). Moreover, a horizontal gradient of sea level pressure enhanced by the reduced sea ice strengthened the surface winds. These results suggested that the reduction of sea ice is the most important factor responsible for the positive ${P}_{\mathrm{w}}$ trend over the SO during winter.

## Introduction

Ocean surface waves can both serve as a climate proxy^1^ and contribute to the atmosphere–ocean interactions at the climate scale^2,3^. Knowledge of long-term signals of ocean surface waves is important for efficient management of coastal communities^4^ and offshore operations^5^. Thus, as climate change impacts are becoming increasingly prominent, long-term changes in ocean surface waves have received increasing attention^1,6–8^.

**Figure 1:**
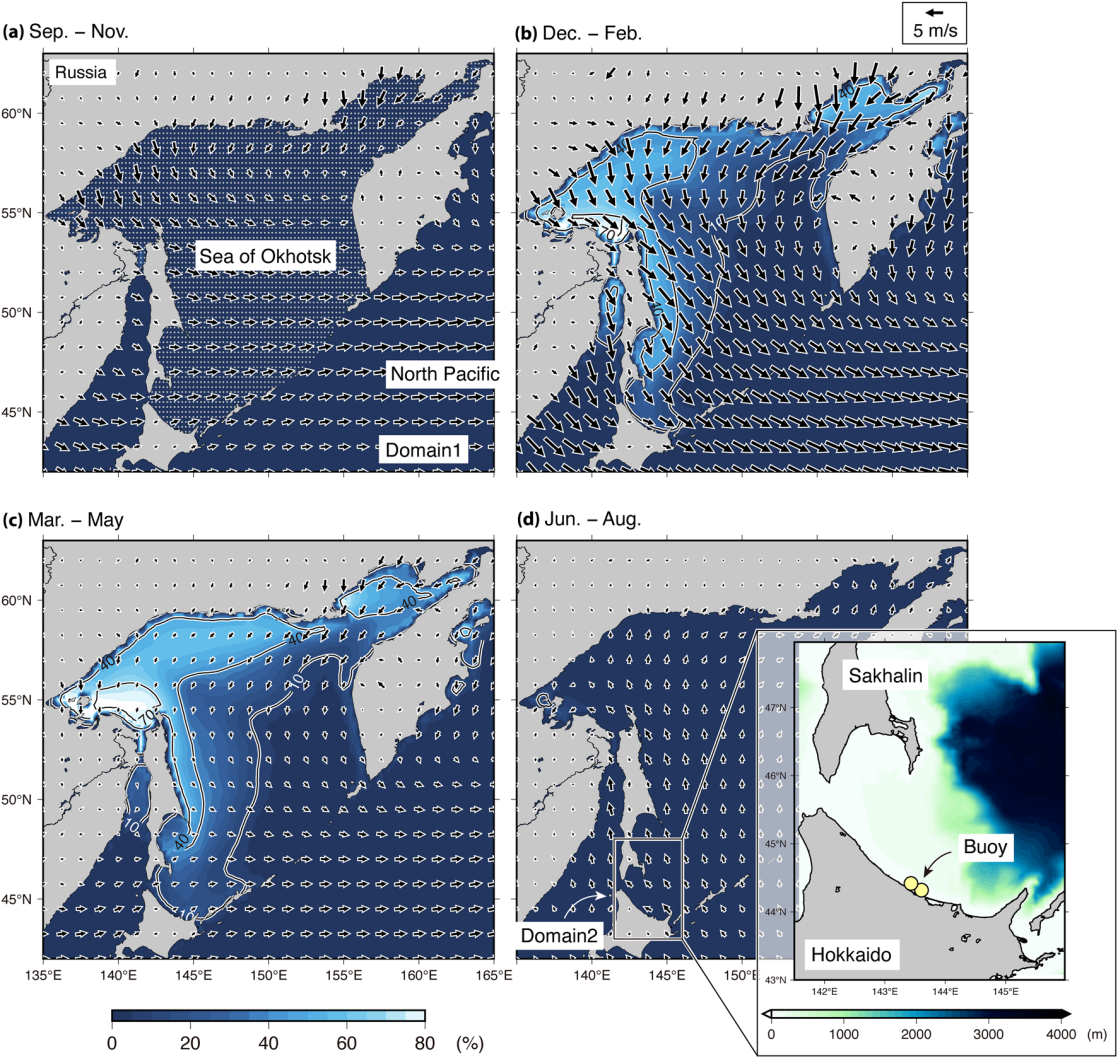
Climatological ice concentration data (color with contour) and surface wind fields (vectors) for (**a**) autumn (September–November), (**b**) winter (December–February), (**c**) spring (March–May), and (**d**) summer (June–August) of 40 years after incorporating two model domains. The surface wind and ice concentration derived from Japanese-55 Reanalysis (JRA55) and National Oceanic and Atmospheric Administration Optimum Interpolation (NOAA OI), respectively. Only the contour of ice concentration was smoothed using a horizontal width of 50 km. (**a**) The stippled area denotes the Sea of Okhotsk. The wind vectors were scaled as shown in the upper right corner of (**b**). **(d**) The right figure is an area of Domain 2 with two buoy locations (yellow circles) and bottom topography (color).

The Sea of Okhotsk (SO) is known as a seasonal sea ice region, where sea ice appears during winter (December–February), after which it gradually starts receding (Fig. 1). In general, sea ice suppresses the wave‒wind interaction because the fetch is reduced as it covers the sea. Moreover, sea ice acts as a physical barrier that modifies the wave dispersion relationship; consequently, the wave energy is attenuated due to conservative scattering and non-conservative dissipation phenomena^9^. Simulating Waves Nearshore (SWAN^10^) and WAVEWATCH III (WW3^11^) are third-generation spectral wave models developed based on the radiative transfer equation for global and regional wave forecasts; additionally, wave–ice parameterization models have been implemented for sea ice-induced wave dissipation. Recent studies investigated the quantitative impacts of sea ice on wave fields using these model simulations with and without sea ice in the marginal ice zones, such as in the Barents Sea and the Gulf of Bothnia^12^, SO^13^, and Indian Ocean^14^, ranging from one storm event to seasonal variations. Additionally, Iwasaki^15^ reported that sea ice significantly reduced the climatological mean and inter-annual variability (standard deviation) of wave power (${P}_{\mathrm{w}}$) by approximately 50% (> 50% in the northwestern area) over the SO during late winter (February–March). Although the extent of sea ice in the SO largely varies annually^16^, Japan Meteorological Agency (JMA) has reported that its maximum value has been decreasing at a rate of 3.5% per decade (https://www.data.jma.go.jp/gmd/kaiyou/english/seaice_okhotsk/series_okhotsk_e.html). Thus, there is a great concern that ocean surface waves will increase due to the decrease in sea ice in SO. However, to the best of our knowledge, previous studies have not yet focused on the SO and not evaluated the long-term trends of ocean surface waves, although several studies have been conducted in the Arctic Sea^17–20^.

Therefore, the present study aimed to evaluate the long-term trends of wave fields in the SO for 40 years and reveal the quantitative contributions of the surface winds and sea ice to the long-term trends. Recently, rather than wave height, ${P}_{\mathrm{w}}$ is also attracting attention as an indicator of the long-term behavior of the wave condition because it represents wave energy accumulated over different months, seasons, and years^15,21–24^. For example, coastal inundation, erosion, and flooding depend not only on the wave height but also on wave period^25^. Therefore, the present study focused on ${P}_{\mathrm{w}}$ as an indicator of the long-term trends of ocean surface waves and used the model simulations of 40 years obtained from WW3 based on three reanalysis products and one satellite-derived sea ice product (see Methods for the model setup and forcing data; Table 1 for data products). Moreover, before investigating the long-term trends of ${P}_{\mathrm{w}}$, the model results were evaluated using the observations of two buoys on the north coast of Hokkaido (Fig. 1d for the buoy positions; see Methods for the validation method).

**Table 1:** List of data products used in this study.

	JRA55	ERA5	MERRA2	NOAA OI
Variables	Wind	Wind, Sea ice	Wind, Sea ice	Sea ice
Temporal reso	6 hourly	Hourly	Hourly	Daily
Spatial reso	0.5625° × 0.5625°	0.25° × 0.25°	0.5° × 0.625°	0.25° × 0.25°
References	Kobayashi et al.^26^	Hersbach et al.^29^	Gelaro et al.^30^	Reynolds et al.^27^, Huang et al.^28^

Three wave model simulations were performed as the control (CTL) run (top part of Table 2). The first model simulation was obtained from WW3 using reanalysis surface wind data from 55-year JMA Reanalysis (JRA55)^26^ and a satellite-derived sea ice product from the National Oceanic and Atmospheric Administration Optimum Interpolation (NOAA OI) sea surface temperature version 2.1^27,28^ (hereafter denoted as JRA55-CTL). In addition to using JRA55-CTL, other two-wave model simulations were performed from the WW3 using surface wind and sea ice products acquired from the European Centre of Medium-range Weather Forecast Reanalysis-5 (ERA5)^29^ (hereafter denoted as ERA5-CTL) and the Modern-Era Retrospective Analysis for Research and Applications (MERRA2)^30^ of National Aeronautics and Space Administration (hereafter denoted as MERRA2-CTL). Additionally, as simulations removed the effect of the long inter-annual time scale of sea ice on wave fields, three simulations were performed (denoted as the Clim-ICE run) (bottom part of Table 2). As shown in Table 2, the three Clim-ICE runs were conducted using the surface wind of original temporal resolution (the same as that of the three CTL runs) and climatological sea ice data. Hereafter, the Clim-ICE experiments that used the JRA55 surface wind and NOAA OI sea ice products, ERA5 surface wind and sea ice products, and the MERRA2 surface wind and sea ice products have been denoted as JRA55-Clim-ICE, ERA5-Clim-ICE, and MERRA2-Clim-ICE, respectively.

**Table 2:** Six model simulations based on the WW3.

Wave hindcast with WW3	Wind	Sea ice
CTL run		
JRA55-CTL*	JRA55	NOAA OI
ERA5-CTL	ERA5	ERA5
MERRA2-CTL	MERRA2	MERRA2
Clim-ICE run		
JRA55-Clim-ICE*	JRA55	NOAA OI (Daily climatology)
ERA5-Clim-ICE	ERA5	ERA5 (Hourly climatology)
MERRA2-Clim-ICE	MERRA2	MERRA2 (Hourly climatology)

*Note that only JRA55-CTL and JRA55-Clim-ICE runs used the model results from Iwasaki^15^.

## Results and discussion

### Validation of the model wave field

**Figure 2:**
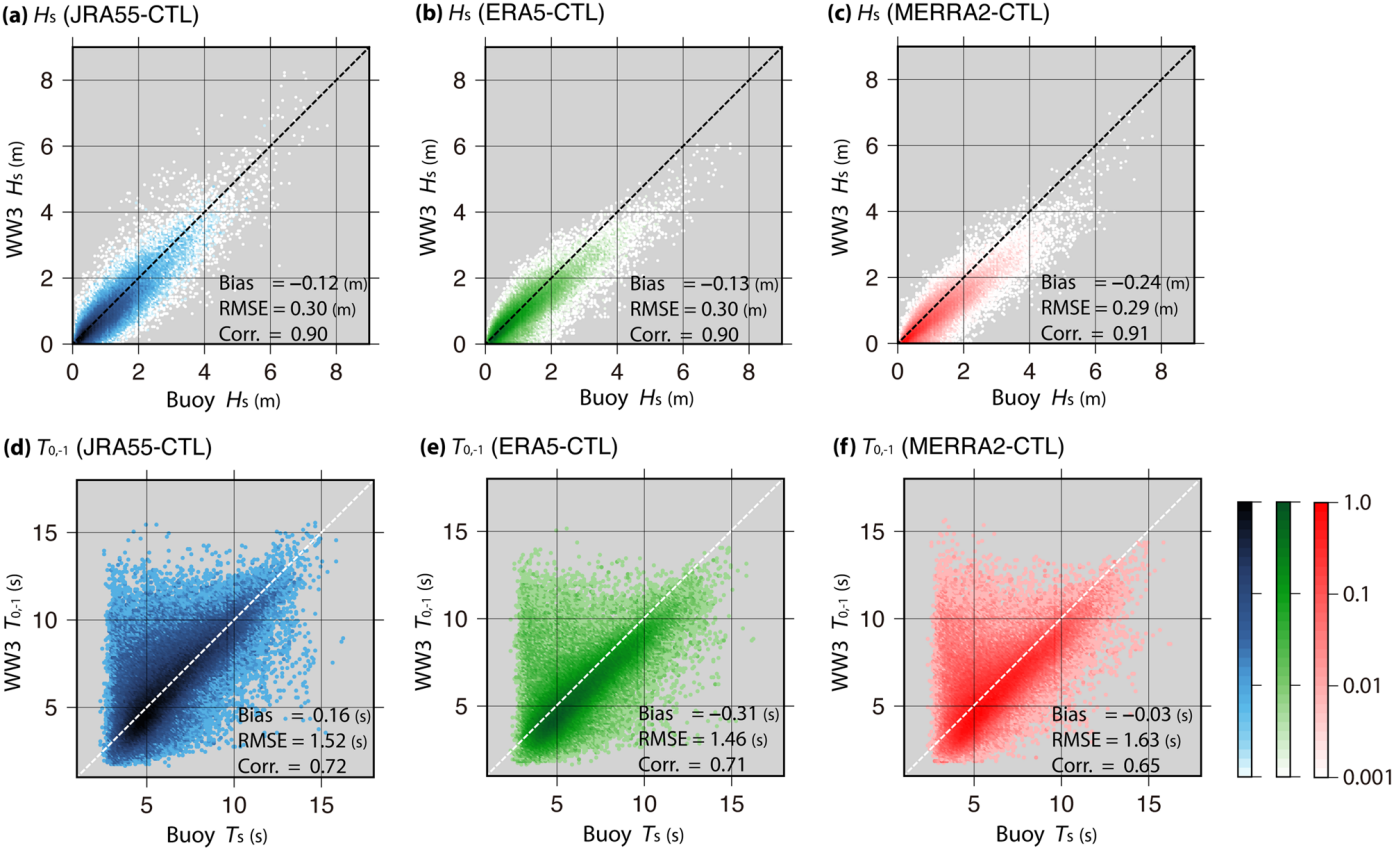
Comparison of (top) ${H}_{\mathrm{s}}$ and (bottom) ${T}_{0,-1}$ values acquired through buoy observations and model simulations: (**a**, **d**) JRA55-CTL (blue), (**b**, **e**) ERA5-CTL (green), and (**c**, **f**) MERRA2-CTL (red). Color shading indicates normalized data density on a log_10_-scale. The number of validation data points (assessed every 2 h) was 115,188. The black or white broken line *y* = *x* was added to each panel. Note that (**a**) and (**d**) present the results published in Iwasaki^15^; however, the results of the buoy observations conducted in 2020 (Monbetsu south) are included here.

The significant wave height (${H}_{\mathrm{s}}$) and wave period (${T}_{0,-1}$) values simulated by the three CTL runs (JRA55-CTL, ERA5-CTL, and MERRA2-CTL) were evaluated using buoy observations. The simulated ${H}_{\mathrm{s}}$ and ${T}_{0,-1}$ were compared to the observed ${H}_{\mathrm{s}}$ and significant wave period (${T}_{\mathrm{s}}$), respectively. Figure 2 shows the comparison results for the model simulations and hourly observations of the two buoys. Table 3 lists the statistical analysis results between the model simulations and buoy observations for the monthly mean. Iwasaki^15^ has previously reported that the JRA55-CTL results are consistent with buoy observations (especially for ${H}_{\mathrm{s}}$) (Fig. 2a, d). In the present study, although the simulated ${H}_{\mathrm{s}}$ and ${T}_{0,-1}$ values of MERRA2-CTL and ERA5-CTL, respectively, were slightly underestimated (especially for ${T}_{0,-1}$ of ERA5-CTL and ${H}_{\mathrm{s}}$ of MERRA2-CTL), compared to the other simulated values, the two model results were simulated with high accuracy similar to JRA55-CTL. Additionally, the errors (except the bias) of the model results for the monthly data were less than those for the two hourly data (Table 3). Regarding the monthly mean, the RMSE of ${H}_{\mathrm{s}}$ and ${T}_{0,-1}$ derived from all simulations were within ~ 0.2 m and ~ 1 s, respectively.

**Table 3:** Statistical values of ${H}_{\mathrm{s}}$ and ${T}_{0,-1}$ between the three model simulations (CTL runs) and buoy observations for monthly mean data.

	JRA55-CTL	ERA5-CTL	MERRA2-CTL
$$H_{{\text{s}}}$$			
Bias (m)	− 0.12	− 0.15	− 0.25
RMSE (m)	0.13	0.16	0.14
Corr	0.93	0.91	0.93
$$T_{0, - 1}$$			
Bias (s)	0.17	− 0.23	0.06
RMSE (s)	0.63	0.87	0.92
Corr	0.86	0.71	0.67

The number of validation data points was 351.

### Trends of wave power

**Figure 3:**
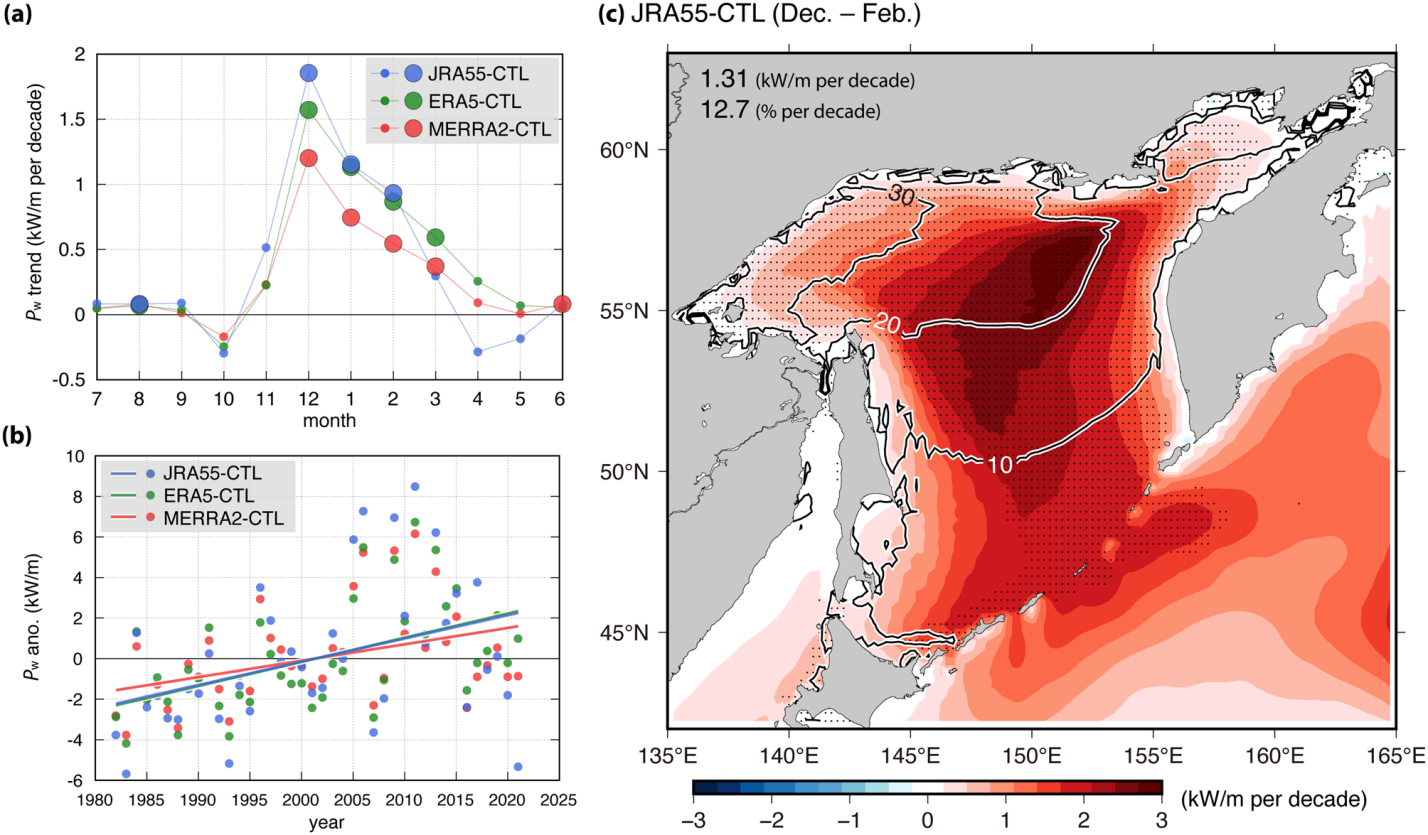
Linear trend of ${P}_{\mathrm{w}}$ from the three CTL simulations over the SO during 40 years. (**a**) Monthly trend of ${P}_{\mathrm{w}}$ averaged over the SO (i.e., stippled area in Fig. 1a). The big circles with black outline indicate significant values (90% confidence level). (**b**) Inter-annual ${P}_{\mathrm{w}}$ anomalies and the linear trend line (significant at the 95% confidence level) averaged over the SO during winter (December–February). Note that the linear trend line of ERA5-CTL (green) overlaps with that of JRA55-CTL (blue) in (**b**). (**c**) Spatial distribution of the ${P}_{\mathrm{w}}$ linear trend during winter derived from JRA55-CTL. The contour indicates percent change (% per decade), defined as (linear trend of ${P}_{\mathrm{w}}$/climatological ${P}_{\mathrm{w}}$) × 100. Black points represent statistical significance at the 95% confidence level. ${P}_{\mathrm{w}}$ linear trends (in units of [kWm^–1^ per decade] and [% per decade]; i.e., the blue trend line in **b**) averaged over the SO during winter, are shown in the top left of (**c**).

The long-term trends of ${P}_{\mathrm{w}}$ in the SO were investigated using the model results of Domain 1 for three CTL experiments (Fig. 3). Although the spatial resolution between the two domains differed, the model results (${P}_{\mathrm{w}}$, ${H}_{\mathrm{s}}$, and ${T}_{0,-1}$) of both domains corresponded to each other, regardless of the model experiments (not shown). Further, the long-term trends were calculated using linear regression, and the significance of the trends was checked using the Mann-Kenndall test^31^. For all simulations, significantly positive trends of ${P}_{\mathrm{w}}$ were observed during winter (December–February) (especially in the JRA55-CTL and ERA5-CTL runs) (Fig. 3a, b). Although the positive trends of ${P}_{\mathrm{w}}$ for JRA55-CTL were observed throughout the SO during winter, they were remarkable especially in the northern areas (Fig. 3c). Additionally, the spatial patterns of the ${P}_{\mathrm{w}}$ trend for JRA55-CTL were consistent with those for the other two simulations (refer Supplementary Fig. S1 for the other two CTL runs). The linear trend values and their ratio over a decade averaged throughout the SO were 1.31 kWm^–1^ and 12.7%, 1.19 kWm^–1^ and 15.3%, and 0.83 kWm^–1^ and 12.1% for JRA55-CTL, ERA5-CTL, and MERRA2-CTL, respectively. Additionally, the increase in ${P}_{\mathrm{w}}$ during winter was also confirmed by the buoy observations in coastal areas (gray line in Supplementary Fig. S2a). Moreover, the model results for the three CTL experiments also suitably reproduced and verified the increase in ${P}_{\mathrm{w}}$ (lines other than gray in Supplementary Fig. S2a). The position of the two buoys (Monbetsu and Monbetsu south) differed slightly by ~ 17 km. However, the ${P}_{\mathrm{w}}$ difference between both positions was much smaller than the increase in ${P}_{\mathrm{w}}$ (Supplementary Fig. S2b). Thereafter, the present study focused on winter showing a significant increase in ${P}_{\mathrm{w}}$.

**Figure 4:**
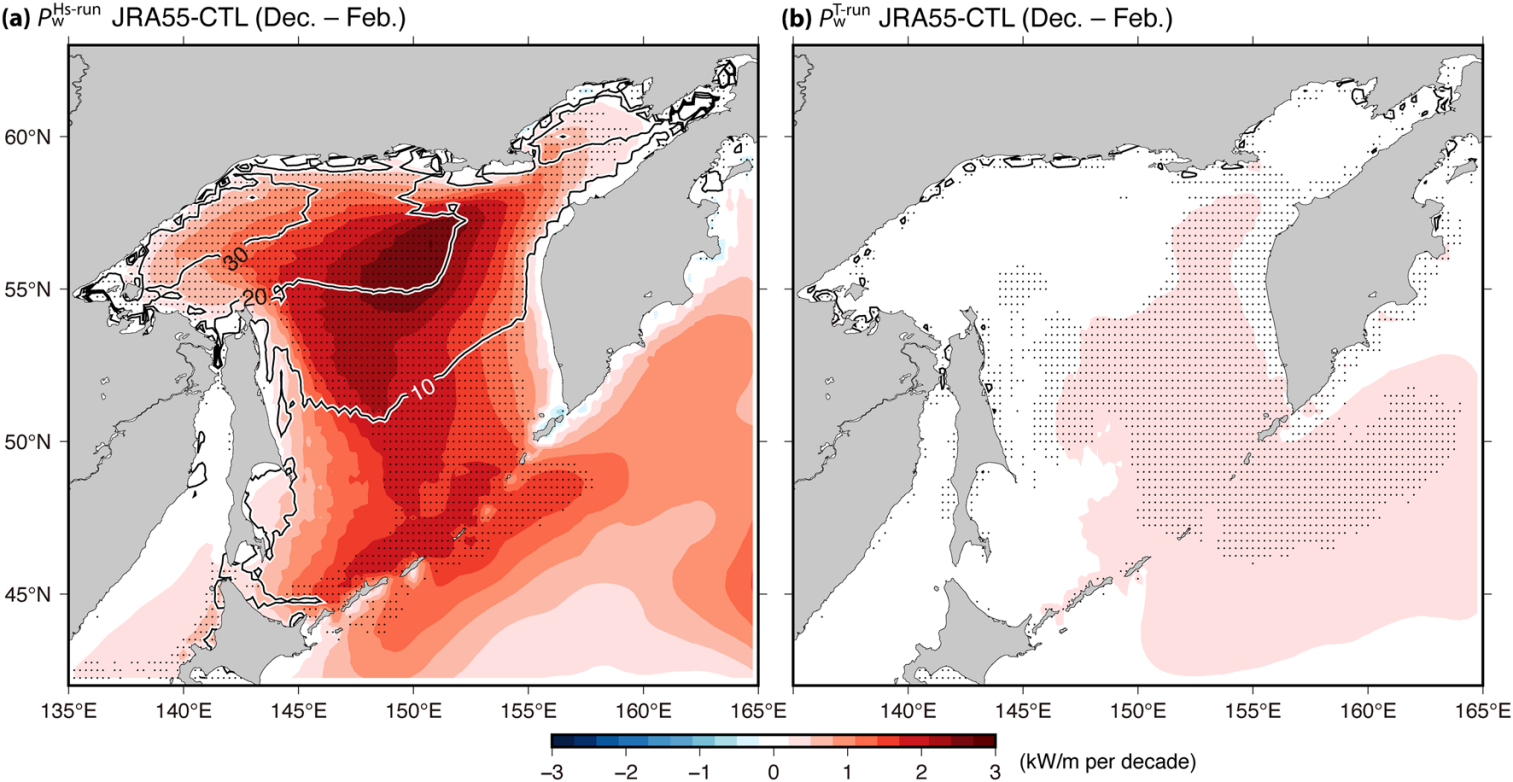
Same as Fig. 3c, but for (**a**) ${P}_{\mathrm{w}}^{\mathrm{Hs}-\mathrm{run}}$ and (**b**) ${P}_{\mathrm{w}}^{\mathrm{T}-\mathrm{run}}$ derived from JRA55-CTL.

${H}_{\mathrm{s}}$ was most likely responsible for the ${P}_{\mathrm{w}}$ trend because ${P}_{\mathrm{w}}$ is mathematically related to the square of ${H}_{\mathrm{s}}$ while it is proportionately related to ${T}_{0,-1}$, as shown in Eq. (1). To assess the quantitative contributions of ${H}_{\mathrm{s}}$ and ${T}_{0,-1}$ to the ${P}_{\mathrm{w}}$ trend, two cases of ${P}_{\mathrm{w}}$ were simply calculated for the three CTL experiments using Eq. (1) by applying an approach similar to that used by Iwasaki^15^. Here, ${P}_{\mathrm{w}}^{\mathrm{Hs}-\mathrm{run}}$ was defined by using the monthly ${H}_{\mathrm{s}}$ and 40-year climatological monthly ${T}_{0,-1}$, and the ${P}_{\mathrm{w}}^{\mathrm{T}-\mathrm{run}}$ was correspondingly obtained by using the 40-year climatological monthly ${H}_{\mathrm{s}}$ and monthly ${T}_{0,-1}$. The ${P}_{\mathrm{w}}^{\mathrm{Hs}-\mathrm{run}}$ and ${P}_{\mathrm{w}}^{\mathrm{T}-\mathrm{run}}$ trends were considered to be the contribution of ${H}_{\mathrm{s}}$ and ${T}_{0,-1}$ to the ${P}_{\mathrm{w}}$ trend, respectively. In fact, the spatial patterns of the ${P}_{\mathrm{w}}$ trend corresponded with those of ${P}_{\mathrm{w}}^{\mathrm{Hs}-\mathrm{run}}$ rather than of ${P}_{\mathrm{w}}^{\mathrm{T}-\mathrm{run}}$, regardless of the simulations (Fig. 4; JRA55-CTL run and Supplementary Fig. S3; other two CTL runs).

### Contributions of wind and sea ice

The positive trend of ${P}_{\mathrm{w}}$ (i.e., increase in ${H}_{\mathrm{s}}$) may be caused by the increase in the surface wind and the reduction in the sea ice. In fact, during winter, surface wind and sea ice area showed positive and negative trends, respectively, for all data products (Table 4). Particularly, the linear trends for surface wind of JRA55 and ice area of ERA5 for a decade were 0.27 ms^–1^ and – 4.65 × 10^4^ km^2^, respectively, which were remarkable compared with other products.

**Table 4:** Linear trends of surface wind (ms^-1^ per decade) and ice area (× 10^4^ km^2^ per decade) averaged over the Sea of Okhotsk for winter during the study period of 40 years.

	JRA55, NOAA OI	ERA5	MERRA2
Wind	0.27 (3.21)	0.17 (2.11)	0.14 (1.73)
Sea ice area	− 1.26 (− 3.52)	− 4.65 (− 10.73)	− 2.45 (− 6.79)

Values in parentheses indicate the relative trends (% per decade).

**Figure 5:**
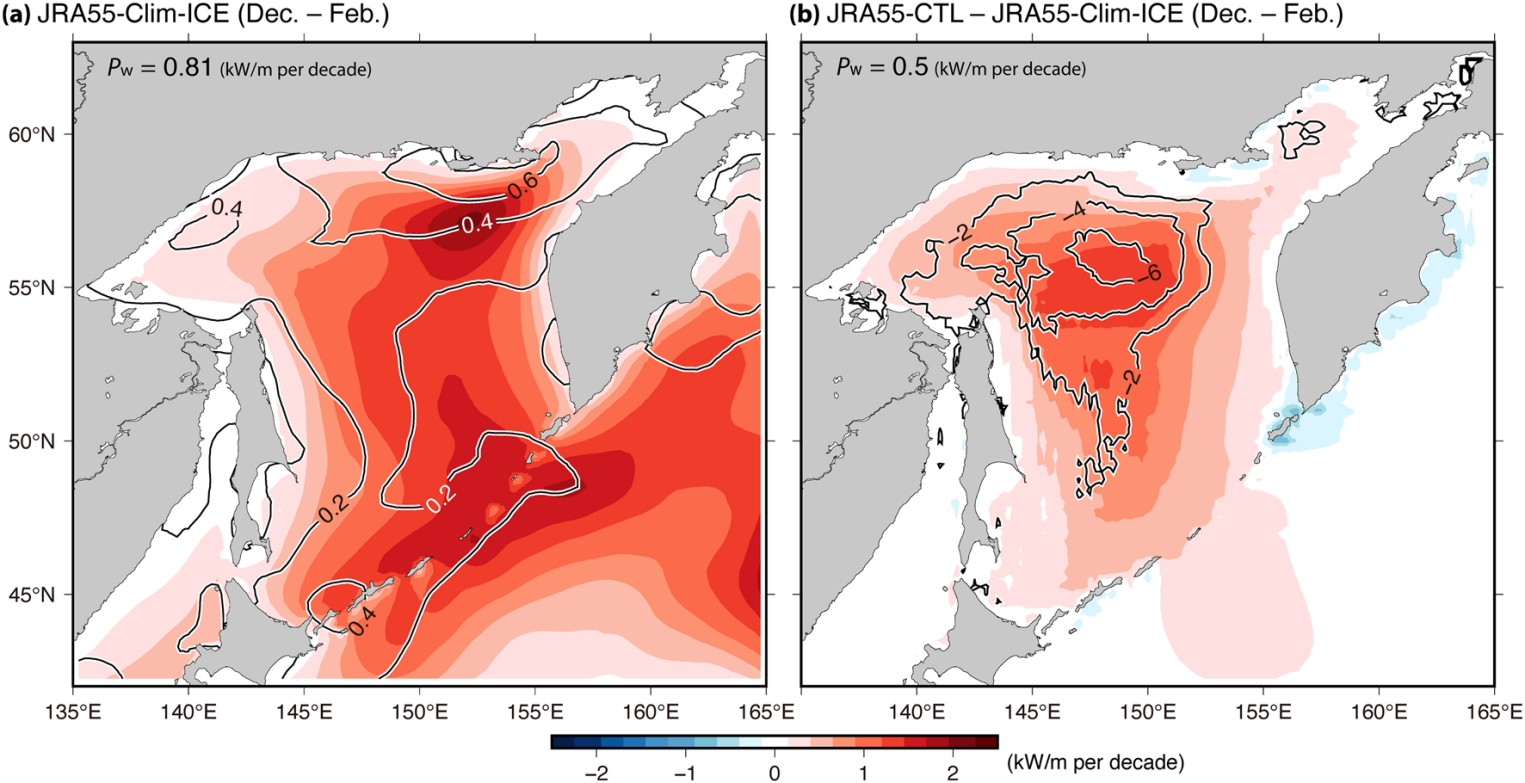
Spatial maps of the ${P}_{\mathrm{w}}$ trend during winter derived from the (**a**) JRA55-Clim-ICE run and (**b**) difference between the JRA55-CTL and JRA55-Clim-ICE runs (i.e., the difference between the colors shown in Figs. 3c and 5a). Contours denote linear trends for (**a**) JRA55 surface wind speed (ms^–1^ per decade) and (**b**) NOAA OI ice concentration (% per decade). The linear trend values of ${P}_{\mathrm{w}}$ averaged over the SO during winter are shown in the top left corner of each panel.

According to the method by Iwasaki^15^, the contributions of surface wind to the ${P}_{\mathrm{w}}$ trend were derived from the Clim-ICE run (i.e., long-term trends of sea ice were eliminated), while the contributions of sea ice were derived from the difference between the CTL and Clim-ICE runs. The spatial distribution of the ${P}_{\mathrm{w}}$ trends for Clim-ICE and for the difference between CTL and Clim-ICE experiments showed common patterns for all simulations. Positive ${P}_{\mathrm{w}}$ trends were observed for Clim-ICE and for the difference (i.e., the CTL—Clim-ICE runs) over the entire SO (Fig. 5, JRA55 simulations; and Supplementary Fig. S4, ERA5 and MERRA2 simulations). Particularly, remarkable positive trends for ${P}_{\mathrm{w}}$ from the Clim-ICE runs (i.e., the contribution of surface wind) were observed in the northern part of the SO. Additionally, the ${P}_{\mathrm{w}}$ difference (i.e., the contribution of sea ice) significantly increased over slightly north of the central part of the area. Moreover, the spatial patterns of the ${P}_{\mathrm{w}}$ trends for the Clim-ICE and for the difference corresponded well with the increasing region of surface wind and reducing region of sea ice, respectively (i.e., contour and color of Fig. 5 and Supplementary Fig. S4) (especially in the trends for the ice concentration and ${P}_{\mathrm{w}}$ difference). The spatial distribution simulations of the ${P}_{\mathrm{w}}$ trends for the Clim-ICE and for the difference showed common patterns while the quantitative contribution of the surface wind and sea ice to the ${P}_{\mathrm{w}}$ trends depended on the simulations. Overall, the ERA5 and MERRA2 simulations indicated that the decreasing trend of sea ice contributed slightly more to the positive trend of ${P}_{\mathrm{w}}$ than the increasing trend of surface wind, while the JRA55 simulations indicated opposite results (top left values of Fig. 5 and Supplementary Fig. S4). These differences in the contributions of the surface wind and the sea ice in the three simulations were also consistent with the trends of surface wind and sea ice shown in Table 4.

### Modification of winds by sea ice

**Figure 6:**
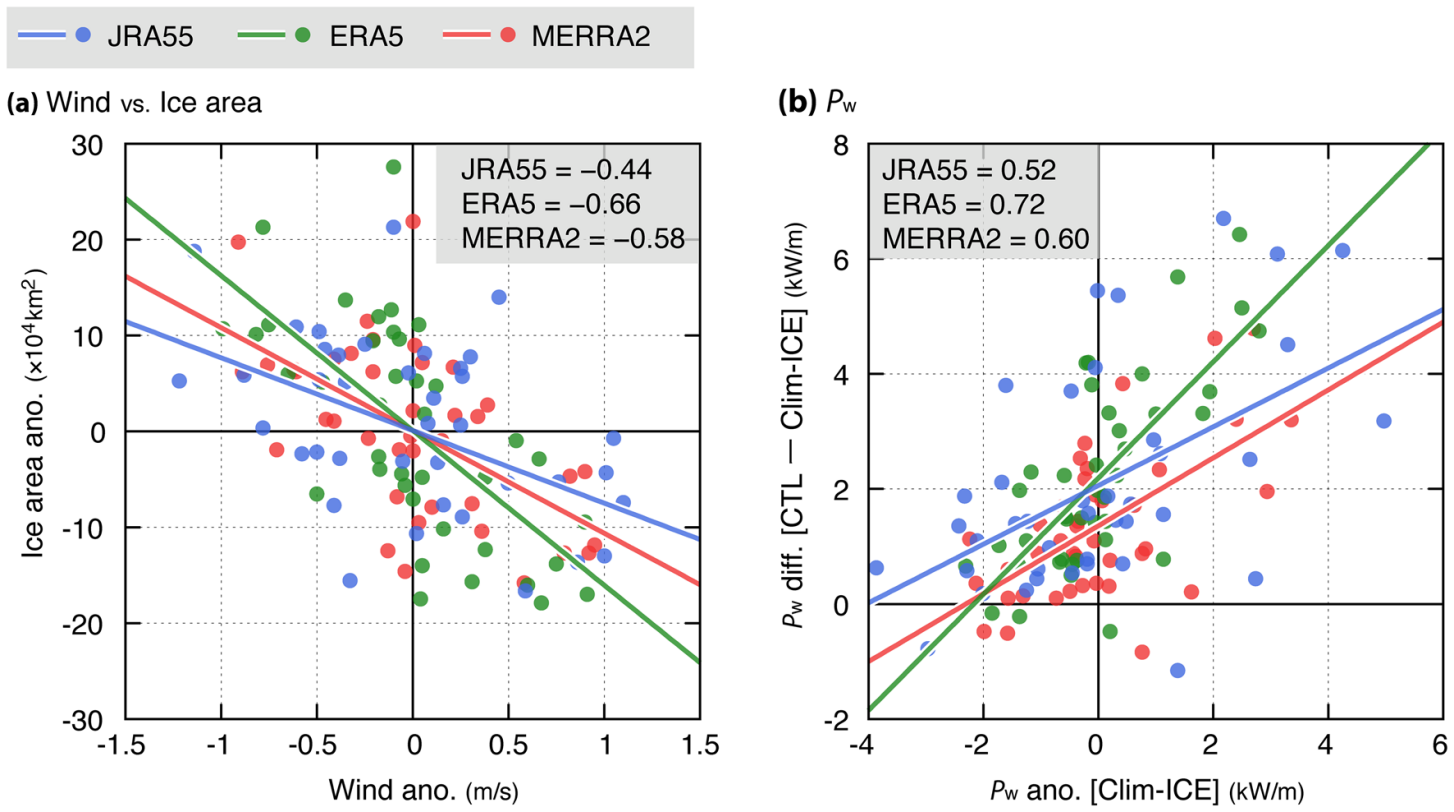
(**a**) Scatter diagram between inter-annual variabilities of surface wind versus ice area anomalies, over the SO during winter. (**b**) As in (**a**), but for the inter-annual variabilities of ${P}_{\mathrm{w}}$ anomalies from the Clim-ICE run versus ${P}_{\mathrm{w}}$ differences between the CTL and Clim-ICE runs. The anomalous values (i.e., inter-annual variabilities of surface wind, ice area, and ${P}_{\mathrm{w}}$) are derived from winter mean minus winter climatological mean. (**a**, **b**) The bold lines indicate the regression line (JRA55, blue; ERA5, green; MERRA2, red). The correlation coefficients (*t*-test, significant at *p* < 0.01) for (**a**) each reanalysis product and (**b**) model simulation are shown in the top corner of each panel (n = 40). (**a**) Note that the correlation coefficients for JRA55 are derived from the JRA55-derived surface wind and NOAA OI-derived ice area anomalies.

**Figure 7:**
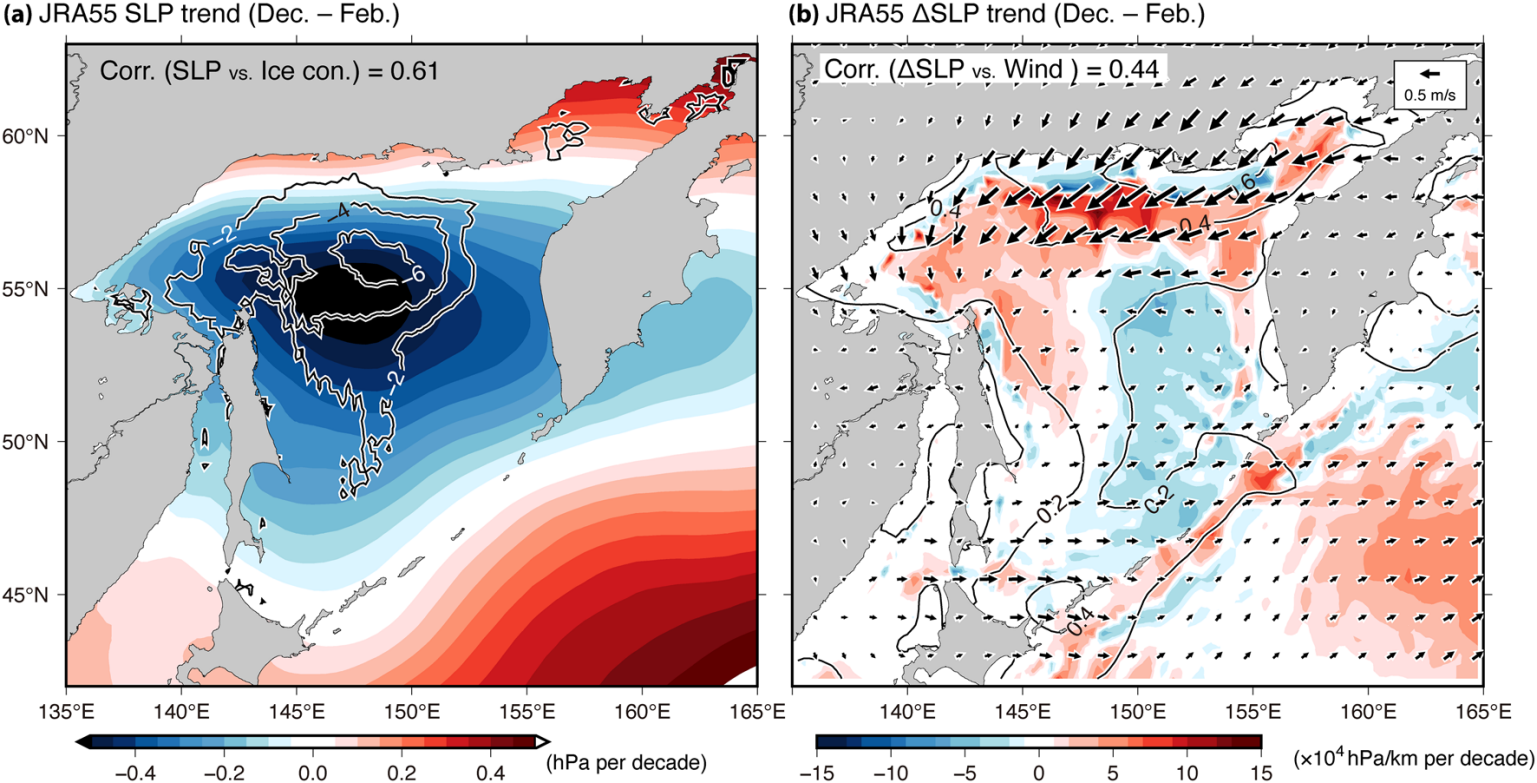
Linear trend maps of (**a**) JRA55 SLP (color) and (**b**) its horizontal gradient (ΔSLP, color) during winter. (**a**) Contours show the linear trend for NOAA OI ice concentration (% per decade) (i.e., same as contour of Fig. 5b). (**b**) The linear trend of JRA55 surface wind field is shown by contours and vectors (ms^–1^ per decade) (same as contour of Fig. 5a except for the vector). The vectors were scaled as indicated in the top right corner of the panel. The correlation coefficients (significant at 99% confidence level according to a *t*-test) for spatial variation of trend values with each grid between (**a**) the ice concentration and SLP, and (**b**) the surface wind and ΔSLP (i.e., between contour and color for each panel) are shown in the top left corner of both panels. Note that the correlation coefficient was calculated only using the grid data over the SO, where the distance from the land was > 100 km and where the climatological ice concentration (NOAA OI ice concentration) during the winter was > 10%.

The decrease in sea ice not only suppresses the wave attenuation but also increases the surface wind speed. In this study, the contributions of surface wind and sea ice to ${P}_{\mathrm{w}}$ trend were evaluated separately; however, Alkama et al.^32^ reported inversion relationships between the sea ice concentration and the near surface wind in the Arctic and Antarctic seas, using six reanalysis products. In fact, during winter, the inter-annual variability of the sea ice, which was defined by the difference of the winter mean from the winter climatological mean, was significantly negatively correlated with that of surface wind (Fig. 6a) for all products, especially ERA5. This negative correlation led to a positive correlation between ${P}_{\mathrm{w}}$ of the Clim-ICE and its difference (CTL—Clim-ICE runs), regardless of the simulations (Fig. 6b). Additionally, the reduced sea ice enhanced the surface wind through the following processes. Since the region experiencing a decrease in the sea ice was relatively warmer than the other regions, a decrease in sea level pressure (SLP) was promoted (Fig. 7a for JRA55-CTL, and Supplementary Fig. S5a and c for ERA5-CTL and MERRA55-CTL). In general, surface wind speeds were associated with the horizontal gradient of SLP (ΔSLP). In fact, for all products, the spatial patterns of the ΔSLP trend were consistent with those of surface wind (Fig. 7b and Supplementary Fig. S5b and d). Furthermore, the correlation coefficient between the surface wind and ΔSLP was 0.44–0.68 (significant with 99% confidence level) for the spatial patterns of the trends (top left values for Fig. 7b and Supplementary Fig. S5b and d). Thus, the reduction in the sea ice may be majorly responsible for the increasing trend of ${P}_{\mathrm{w}}.$

## Conclusions

The present study evaluated the long-term ${P}_{\mathrm{w}}$ trends in the SO during 40 years, based on three wave model simulations using three reanalysis (JRA55, ERA5, and MERRA2) and one satellite sea ice product (NOAA OI). In addition to the three model simulations with original sea ice (i.e., hourly or daily data), three model simulations were also conducted using the climatological sea ice data (i.e., six case simulations in total) to reveal the quantitative contributions of surface winds and sea ice to the long-term trends of ${P}_{\mathrm{w}}$. The model results were validated using the observations of two buoys located in the southern part of the SO and an overall consistency was observed between the model results and the buoy observations, regardless of the products used. All simulations showed the following results: ${P}_{\mathrm{w}}$ showed a significantly positive trend (approximately 12–15% per decade) during winter (December–February) in the SO. This positive ${P}_{\mathrm{w}}$ trend was predominantly caused by the strengthened ${H}_{\mathrm{s}}$. Additionally, on comparing the model simulations with original sea ice data (daily or hourly data) and climatological sea ice data, a positive trend of ${P}_{\mathrm{w}}$ was found to be enhanced by the reduction in the sea ice (reduction of direct wave attenuation due to the sea ice) and the increase in the surface wind. The reduction of sea ice also strengthened the surface wind due to the increase in the horizontal gradient of SLP. The findings of the present study suggested that the reduction in the sea ice is majorly responsible for the increase in ${P}_{\mathrm{w}}$ during winter. Studies that quantitatively examine the effects of surface wind and sea ice separately on long-term wave climate are rare, even if other sea ice areas are included. We believe that the wave–sea ice–atmosphere interaction shown in this study can improve our understanding of past, present, and future wave climates in the marginal ice zone.

## Methods

### Model setup

The details of the WW3 setup were the same as mentioned in Iwasaki^15^, except the reanalysis product used for forcing data. The WW3 setup can be briefly described as follows: The wave simulations were conducted using version 6.07 of the third-generation WW3. The model simulations were conducted for 40 years from September 1, 1981 to the end of August 2021 and two model domains were conducted, Domain 1 (42°–63° N, 135°–165° E; Fig. 1a) and Domain 2 (43°–48° N, 141.5°–146° E; Fig. 1d), using a nesting process. ST6 parameterization was used for wave energy input and dissipation terms^33–35^. For ice source terms, based on the results of Iwasaki and Otsuka^13^, the attenuation rate by Meylan et al.^36^ (denoted as IC4M2 in WW3) was used in the present study. Additionally, a simple diffusive scattering model (denoted as IS1 in WW3) was selected. The frequency space was 0.035–1.1 Hz, which was logarithmically discretized into 30 increments, and the directional resolution was 10°. GEBCO2020 (https://www.gebco.net/data_and_products/gridded_bathymetry_data/gebco_2020) was used to provide the bottom topography and coastlines.

### Forcing fields

Table 1 lists all the forcing data products of the wave model used in this study. In this study, three reanalysis products (the JRA55^26^, the ERA5^29^, and the MERRA2^30^) and one satellite-derived sea ice product (NOAA OI^27,28^) were used for forcing wave model experiments). As shown in Table 2, three control (CTL) (i.e., the JRA55-CTL, the ERA5-CTL, and the MERRA2-CTL) and three Clim-ICE (the JRA55-Clim-ICE, the ERA5-Clim-ICE, and the MERRA2-Clim-ICE) experiments were performed, respectively. NOAA OI ice concentration was used for the JRA55-CTL run because the ice concentration data were not provided by JRA55. Three simulations for the CTL runs were performed using surface wind and ice concentration of original temporal resolution for each product. Comparative assessments performed by previous studies showed that the surface wind of JRA55, ERA5, and MERRA2 were highly consistent with independent observations in the sea ice areas, such as the Arctic^37^ and Antarctic^38^. The three Clim-ICE runs were conducted using hourly climatological data (daily climatological data were used only for JRA55-Clim-ICE) of the ice concentration. The climatology of the sea ice concentration determined using three Clim-ICE runs was derived for the entire computation period (i.e., 40 years). However, the wave simulations of 40 years using the JRA55 surface wind product and NOAA OI sea ice product (i.e., the JRA55-CTL and the JRA55-Clim-ICE runs) represented the model results by Iwasaki^15^.

Further, the wind and sea ice concentration data were linearly interpolated to the same spatial grid in the wave simulation of both domains. Additionally, in this study, the sea ice area indicated the total area of grid points for the sea ice concentration in the SO and not the sea ice extent. Moreover, monthly SLP data from the three reanalysis products (JRA55, ERA5, and MERRA2) were used in this study, although they were not used for wave calculations.

${H}_{\mathrm{s}}$ and ${T}_{0,-1}$ were recorded hourly during the computation period. ${T}_{\mathrm{0,2}}$ and ${T}_{0,-1}$ were defined as ${{T}_{0,\mathrm{n}}=\left({{m}_{0}/m}_{\mathrm{n}}\right)}^{1/\mathrm{n}}$, where, ${m}_{0}$ (${m}_{\mathrm{n}}$) was the order-zero (order-n) spectral moment. Thereafter, monthly averaged ${H}_{\mathrm{s}}$ and ${T}_{0,-1}$ were calculated from the hourly data. ${P}_{\mathrm{w}}$ was derived from the monthly ${H}_{\mathrm{s}}$ and ${T}_{0,-1}$ values, which showed sufficient accuracy (see “Validation of the model wave field” Section for model validation), using the following expression:

1 $$\begin{array}{*{20}c} {P_{{\text{w}}} = \displaystyle\frac{{\rho {\text{g}}^{2} }}{64\pi }H_{{\text{s}}}^{2} T_{0, - 1} ,} \\ \end{array}$$

where $\rho$ (= 1025 kg m^–3^) is the density of seawater and $\mathrm{g}$ (= 9.806 m s^–2^) is the gravitational acceleration.

### Validation method using buoys

To validate the model results, two hourly data of ${H}_{\mathrm{s}}$ and ${T}_{\mathrm{s}}$ for the observations of two buoys were used from the Nationwide Ocean Wave Information Network for Ports and Harbors (NOWPHAS; https://www.mlit.go.jp/kowan/nowphas/index_eng.html), provided by the Ports and Harbors Bureau, Ministry of Land, Infrastructure, Transport, and Tourism. Supplementary Table S1 provides the details of the two buoys (buoy positions are shown in Fig. 1). ${H}_{\mathrm{s}}$ and ${T}_{\mathrm{s}}$ of the NOWPHAS observations were obtained from the zero-up-crossing analysis. Further, according to Goda^39^, the simulated ${H}_{\mathrm{s}}$ and ${T}_{0,-1}$ were compared to the observed ${H}_{\mathrm{s}}$ and ${T}_{\mathrm{s}}$, respectively.

To obtain the simulated values at the buoy positions, the fields were linearly interpolated to the positions using the surrounding four grid values acquired from Domain 2. Statistical parameters of bias, root mean square error (RMSE), and correlation coefficient ($r$) were calculated as follows:

$$ {\text{Bias}} = \overline{{X_{{\text{s}}} }} - \overline{{X_{{\text{o}}} }} , $$

$$ {\text{RMSE}} = \sqrt {\sum (X_{{\text{s}}} - X_{{\text{o}}} - {\text{bias}})^{2} } , $$

$$r = \frac{{\sum (X_{{\text{s}}} - \overline{{X_{{\text{s}}} }} )( {X_{{\text{o}}} - \overline{{X_{{\text{o}}} }} } )}}{{\sqrt {\sum (X_{{\text{s}}} - \overline{{X_{{\text{s}}} }} )^{2} } \sqrt {\sum (X_{{\text{o}}} - \overline{{X_{{\text{o}}} }} )^{2} } }} ,$$

where ${X}_{\mathrm{s}}$ and ${X}_{\mathrm{o}}$ are the simulated and observed values, respectively.

## Supplementary Information

Supplementary Information.

## Data Availability

The data in this study were listed as follows: NOWPHAS buoy (https://www.mlit.go.jp/kowan/nowphas/index_eng.html), JRA55 (https://rda.ucar.edu/datasets/ds628.0), ERA5 (https://www.ecmwf.int/en/forecasts/datasets/reanalysis-datasets/era5), MERRA2 (https://gmao.gsfc.nasa.gov/reanalysis/MERRA-2/), NOAA for OISST version 2.1 (https://psl.noaa.gov/data/gridded/data.noaa.oisst.v2.highres.html).
