# Supplementary material for: Increase in the wave power caused by decreasing sea ice over the Sea of Okhotsk in winter
Source: Sci Rep. 2023 Feb 13;13:2539. doi: 10.1038/s41598-023-29692-9 (PMC9925778; doi:10.1038/s41598-023-29692-9)
Supplement: Supplementary file 1 — Supplementary Information. [file 41598_2023_29692_MOESM1_ESM.pdf]

SUPPLEMENTARY INFORMATION

Increase in the wave power caused by decreasing sea ice over the Sea of Okhotsk in winter

Shinsuke Iwasaki

Civil Engineering Research Institute for Cold Region, Public Works Research Institute,
1–3–1–34, Toyohira, Sapporo 062–8602, Japan

Contents of this file

Figures S1 to S5
Table S1

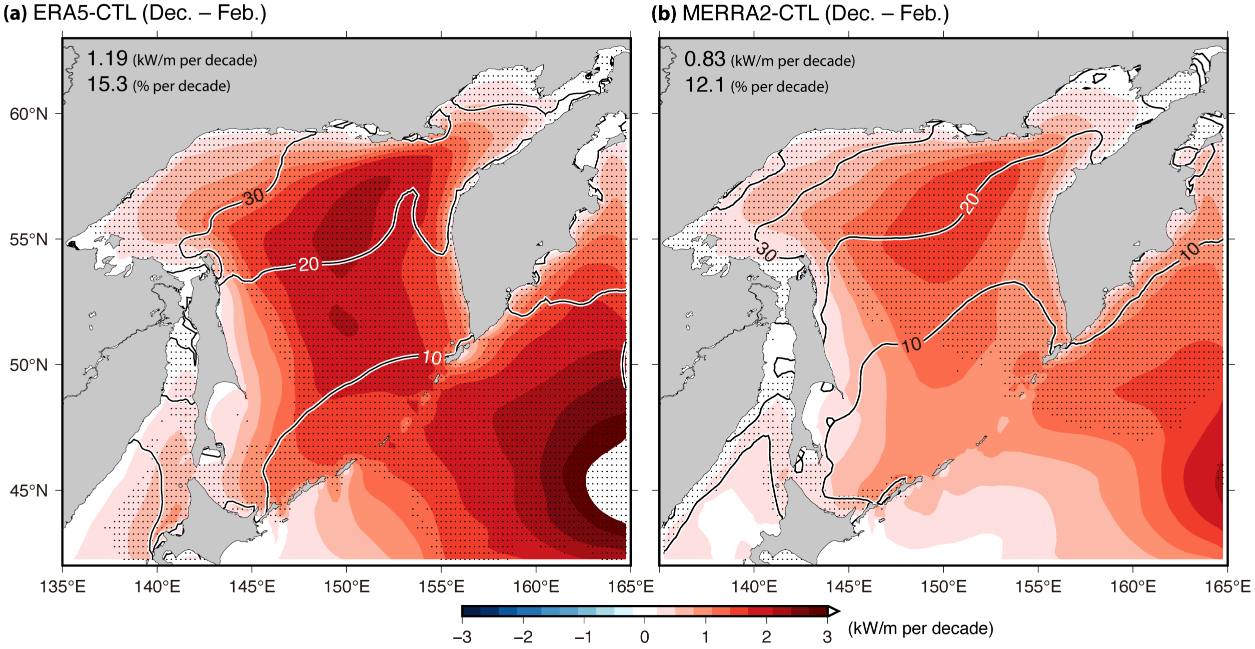

Figure S1. Same as in Figure 3**c**, but for (**a**) European Centre of Medium-range Weather Forecast Reanalysis-5-Control (ERA5-CTL) and (**b**) Modern-Era Retrospective Analysis for Research and Application-2-Control (MERRA2-CTL).

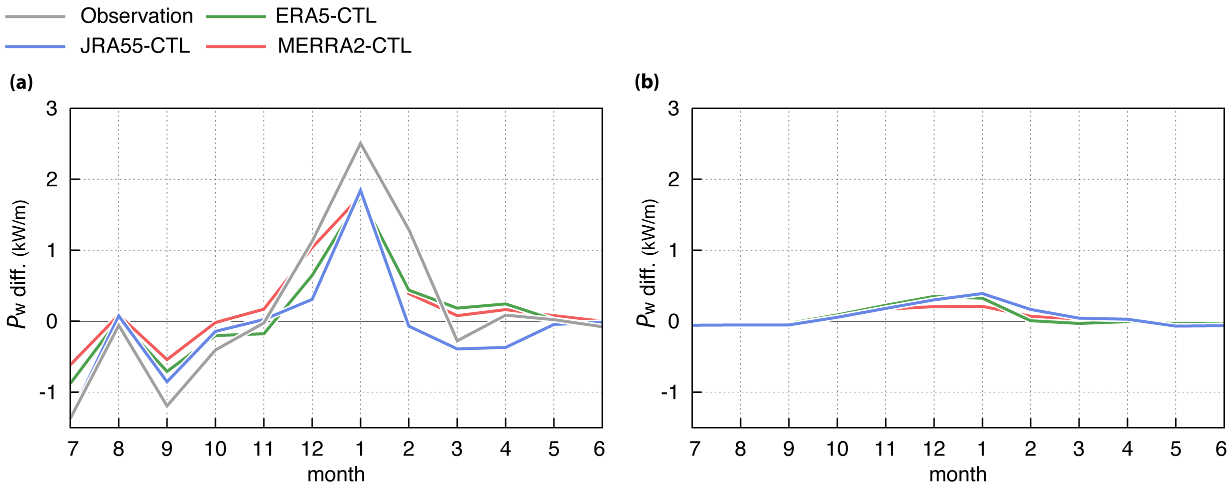

Figure S2. (**a**) Difference in the climatological monthly P_w between two buoys (Monbetsu south and Monbetsu). P_w difference is defined as the difference between the observations of the two buoys. As shown in Table S1, the periods of the two buoy data differed, that is, from the 1980s to the 1990s for Monbetsu and from the 2000s to the 2010s (but including 2020) for Monbetsu south. The values for three CTL runs were used only when there were two-hourly observation data (i.e., data for the validation). (**b**) As in (**a**), but the difference between two buoy positions for climatological monthly P_w data for three CTL runs was derived from the entire computation period (i.e., 40 years). In this figure, in a line with other analyses, P_w was estimated from the monthly H_s and $T_{0,-1}$ (T_s for the buoy). The monthly data points used for the climatological monthly mean data in (**a**) were 178 (173) for the Monbetsu south (Monbetsu) buoy, while 480 data points were used for climatological monthly data for (**b**).

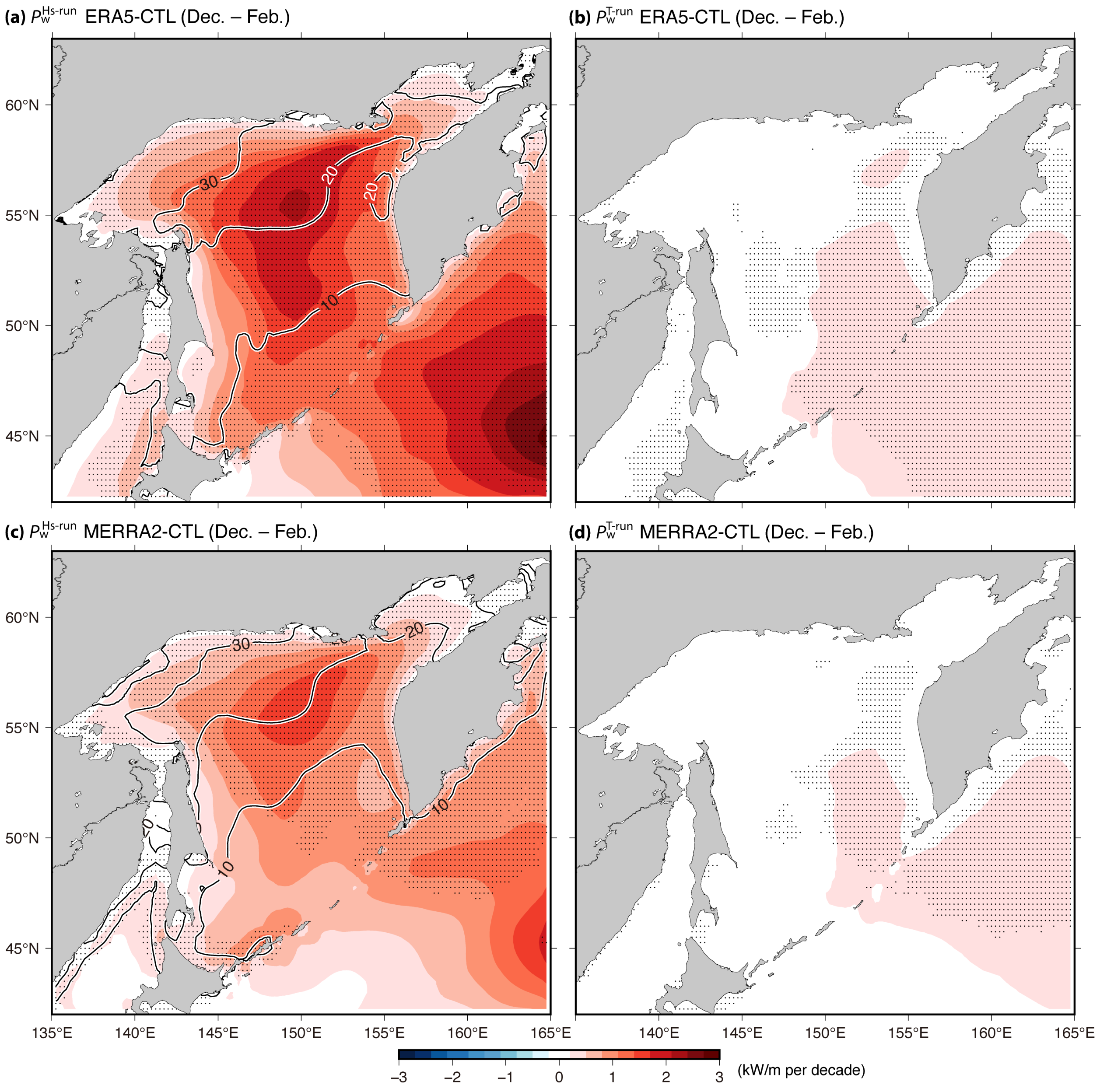

Figure S3. As in Figure 4, but for (**a**, **b**) ERA5-CTL and (**c**, **d**) MERRA2-CTL.

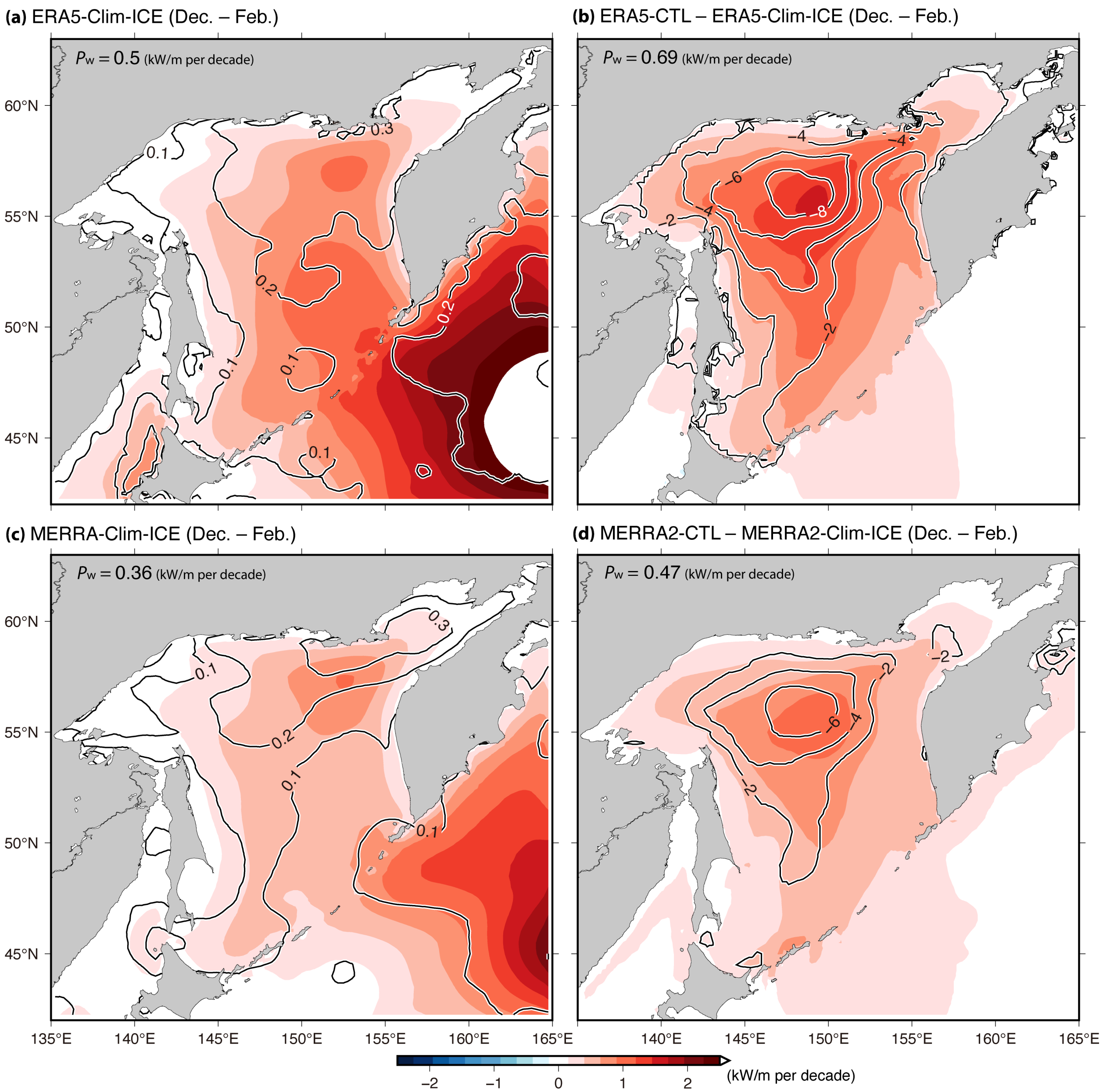

Figure S4. Same as in Figure 5, but for (**a**) ERA5-Clim-ICE run, (**b**) difference between the ERA5-CTL and ERA5-Clim-ICE runs, (**c**) MERRA2-Clim-ICE run, and (**d**) difference between the MERRA2-CTL and MERRA2-Clim-ICE runs. The linear trend values of P_w averaged over the SO during winter for each model simulation are indicated in the top-left corner of each panel.

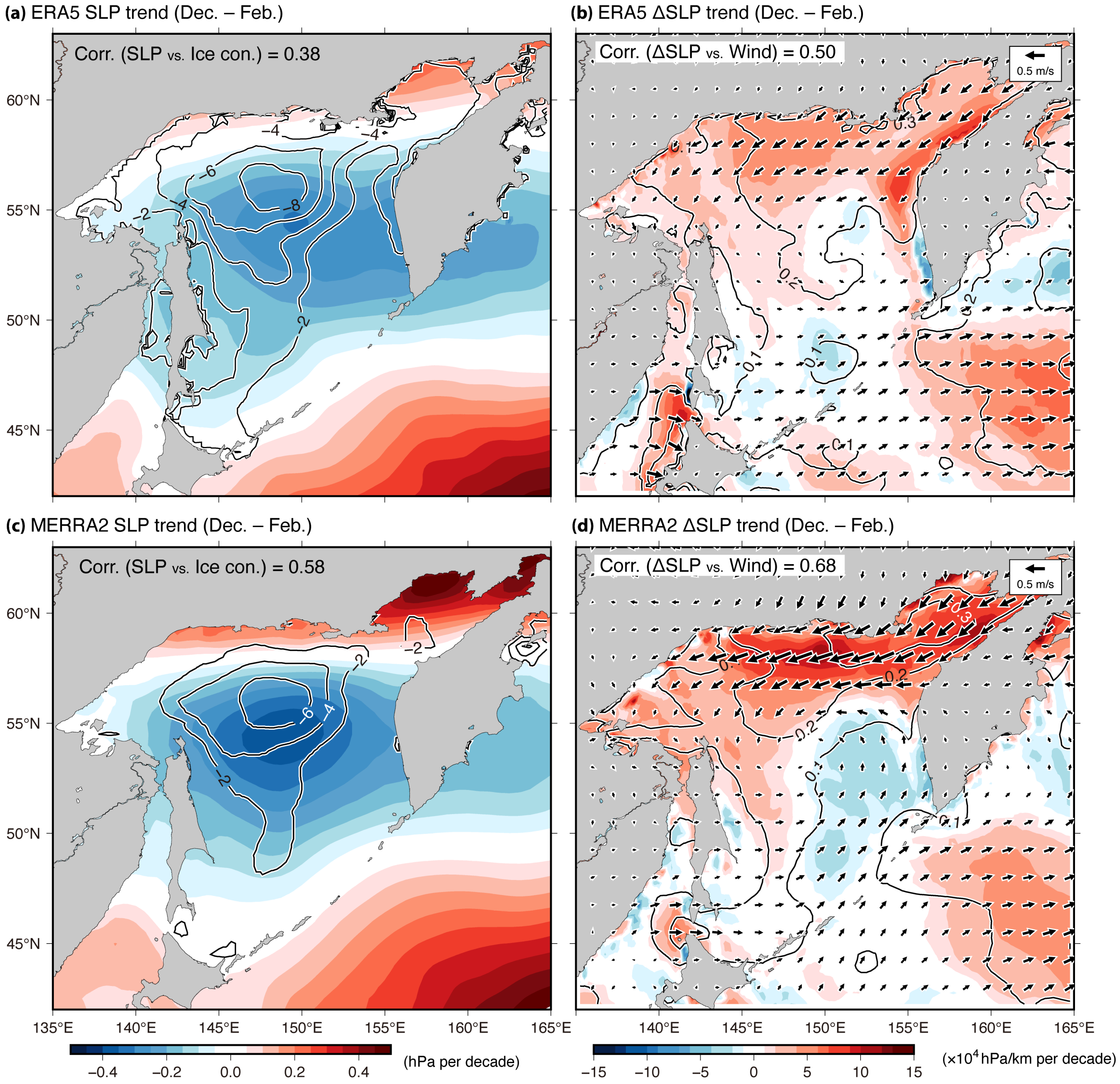

Figure S5. As in Figure 7, but for (**a**, **b**) ERA5 and (**c**, **d**) MERRA2. The scales of SLP and ΔSLP trend values are given below (**c**) and (**d**), respectively. When calculating the correlation coefficient (*t*-test, significant at $p < 0.01$), (**a**, **b**) ERA5 and (**c**, **d**) MERRA2 sea ice concentration data were used to select the grid data (i.e., grids where the climatological ice concentration during winter was > 10%).

Table S1. Details of two buoys used for model validation in this study.

Location name	Location	Used data period (year)	Observation depth (m)	Distance from the coast (m)
Monbetsu	44.416ºN, 143.432ºE	1975−2001	52	9,590
Monbetsu south	44.318ºN, 143.607ºE	2000−2012, 2018−2020	52.6	8,200
